# Effects of supplemental nitrogen application on physiological characteristics, dry matter and nitrogen accumulation of winter rapeseed (*Brassica napus* L.) under waterlogging stress

**DOI:** 10.1038/s41598-020-67260-7

**Published:** 2020-06-23

**Authors:** Shengnan Men, Honglin Chen, Shanghong Chen, Shenghua Zheng, Xueshan Shen, Changtao Wang, Zepeng Yang, Dinghui Liu

**Affiliations:** Water Saving Agriculture Engineering Technology Center, Soil and Fertilizer Research Institute, Sichuan Academy of Agricultural Sciences, Chengdu, 610066 China

**Keywords:** Plant sciences, Plant stress responses, Abiotic

## Abstract

Waterlogging stress is a common limiting factor for winter rapeseed, which greatly affects the growth and potential production. The present study was conducted to investigate the effects of waterlogging with different durations (0day (D0), 6days (D6) and 9days (D9)) and supplemental nitrogen fertilization (N1, 0 kg ha^−1^; N2, 30 kg ha^−1^; N3, 60 kg ha^−1^ and N4, 90 kg ha^−1^) on the physiological characteristics, dry matter and nitrogen accumulation in winter rapeseed (Chuanyou36). The results showed that the supplementary application of nitrogen fertilizer could effectively improve the physiological indexes of winter rapeseed in both pot and field experiments. The supplemental nitrogen increased the chlorophyll content in leaves, enhanced the activities of SOD, CAT, and POD, and decreased the MDA content in leaves and roots of rapeseed. The chlorophyll contents, the antioxidant enzyme activity of leaves and roots significantly increased under D6N3 and D9N4 conditions in both (pot and field) experiments. However, MDA contents significantly decreased compared with waterlogging without nitrogen application. Moreover, the application of nitrogen fertilizer after waterlogging increased the accumulation of dry matter and nitrogen in rapeseed at different growth stages. Therefore, waterlogging stress significantly inhibited the growth and development of rapeseed, but the application of nitrogen fertilizer could effectively reduce the damage of waterlogging. The N-induced increase in waterlogging tolerance of rapeseed might be attributed to the strong antioxidant defense system, maintenance of photosynthetic pigments and the nutrient balance.

## Introduction

The effect of waterlogging on the plant’s growth is a complex phenomenon and varies with the plants species^1–3^, genotypes^4^, developmental stages of plants^5,6^ and the duration and severity of stress^3^. Although water is vital for plants but over water is harmful by lowering the oxygen level in the soil which led to serious consequences of necrosis and stunting^7^. Normally, waterlogging can produce effects on the physiological processes such as decreased the activities of antioxidant^8^ and reduced the photosynthetic performance of crop plants^9^. Moreover, it can inhibit the plant growth by decreasing the accumulation of dry matter and nitrogen content by altering the metabolism and plant nutrient availability^10–12^.

The application of nitrogen fertilizer has significant regulatory effects on the growth and yield of winter rapeseed^13,14^. However, the nitrogen not only improves the crop yield but also act as a stimulating factor to regulate endogenous hormone levels and balance between various hormones to enhance crop resistance against environmental stresses^15^. Previous studies have shown that the application of nitrogen fertilizer can increase the enzyme activities of soluble starch synthase and bound starch synthase and protein content in wheat grains^16^ and it can improve the photosynthetic performance of corn^17^ and increase the production by more than 10% of cotton under waterlogged conditions^18^. In addition, the application of an appropriate amount of nitrogen fertilizer had been found to increase the content of plant dry matter, chlorophyll, photosynthetic rate, and yield of winter rapeseed^19^.

Rapeseed is an important oilseed crop in China which is rapidly expanding as a rotation crop following rice^20^. However, it is sensitive to waterlogging, which usually refers to an adverse environment that limits crop growth and development^21^. Waterlogging frequently occurs to those regions where rainfall is extensive, weak soil drainage and the high-water level varies strongly^22^. Chengdu Plain is one of the main winter rapeseeds producing areas in Sichuan, which is located in the western Sichuan Basin. In the Chengdu Plain, most of the rainfall occurs during the seedling stage of the winter rapeseed. Therefore, the growth and development of rapeseed are susceptible to waterlogging stress during early growth stages.

Previous studies have shown that the rapeseed yield was more dependent on fertilizer application under drought stress^23^. Appropriate application of nitrogen fertilizer can improve rapeseed yield and redistribution of nitrogen^24^, and can alleviate the effect of drought stress on rapeseed growth and development^25^. However, there are few studies on how the application of nitrogen fertilizer under waterlogging stress affects the physiological characteristics, nitrogen uptake and utilization of rapeseed. Therefore, the present study was conducted to assess the effects of nitrogen application on the physiological characteristics, dry matter and nitrogen uptake and utilization of rapeseed under the different duration of waterlogging, to get a better theoretical basis for rational nitrogen fertilizer operation for seasonal waterlogging of rapeseed production (especially rice stubble direct-seeded rapeseed) in the Yangtze River Basin.

## Results

### Photosynthetic pigments

**Figure 1:**
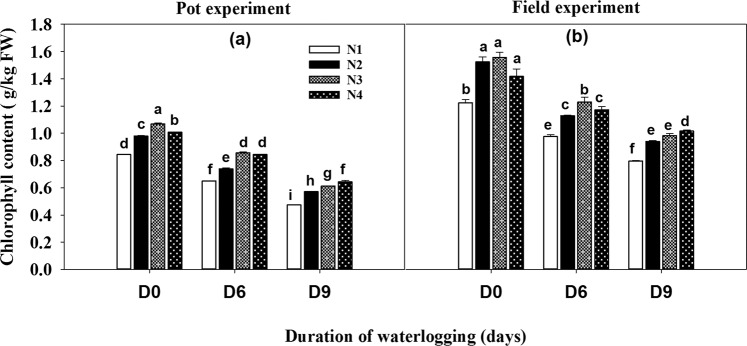
Influence of duration of waterlogging and nitrogen application on the content of chlorophyll of rapeseed leaves under pot and field experiments. Values are means of three replicates ±SE. Small alphabetical letters above mean denote the significant differences among treatment at P ≤ 0.05. N1, 0 kg ha^−1^; N2, 30 kg ha^−1^; N3, 60 kg ha^−1^; and N4,90 kg ha^−1^. D0, refers to without waterlogging; D6, refers to waterlogging for 6 days and D9, refers to waterlogging for 9 days.

When plants were exposed to waterlogging stress conditions at the seedling stage, the total leaf chlorophyll concentration of rapeseed plants was significantly decreased in both pot and field experiments, but the application of nitrogen fertilizer alleviated the plant damage caused by waterlogging (Fig. 1). The nitrogen fertilized plants had higher leaf chlorophyll concentration compared with no application of nitrogen (N1), although the values were still lower than the no waterlogged plants. The most significant effect of nitrogen fertilizer application was observed on the D0N3, D6N3, and D9N4 treatments, with the most significant increased by 26.7, 31.9, and 36.0% in pot experiment and 14.9, 25.9, and 27.5% in field experiment compared with no fertilizer-treated plants. Additionally, the higher content of chlorophyll was recorded in field experiment compared with the pot experiment. Moreover, the concentration of chlorophyll in pot and field experiments was increased with the lower duration of waterlogged stress and the higher level of supplemental nitrogen application.

### Lipid peroxidation

**Figure 2:**
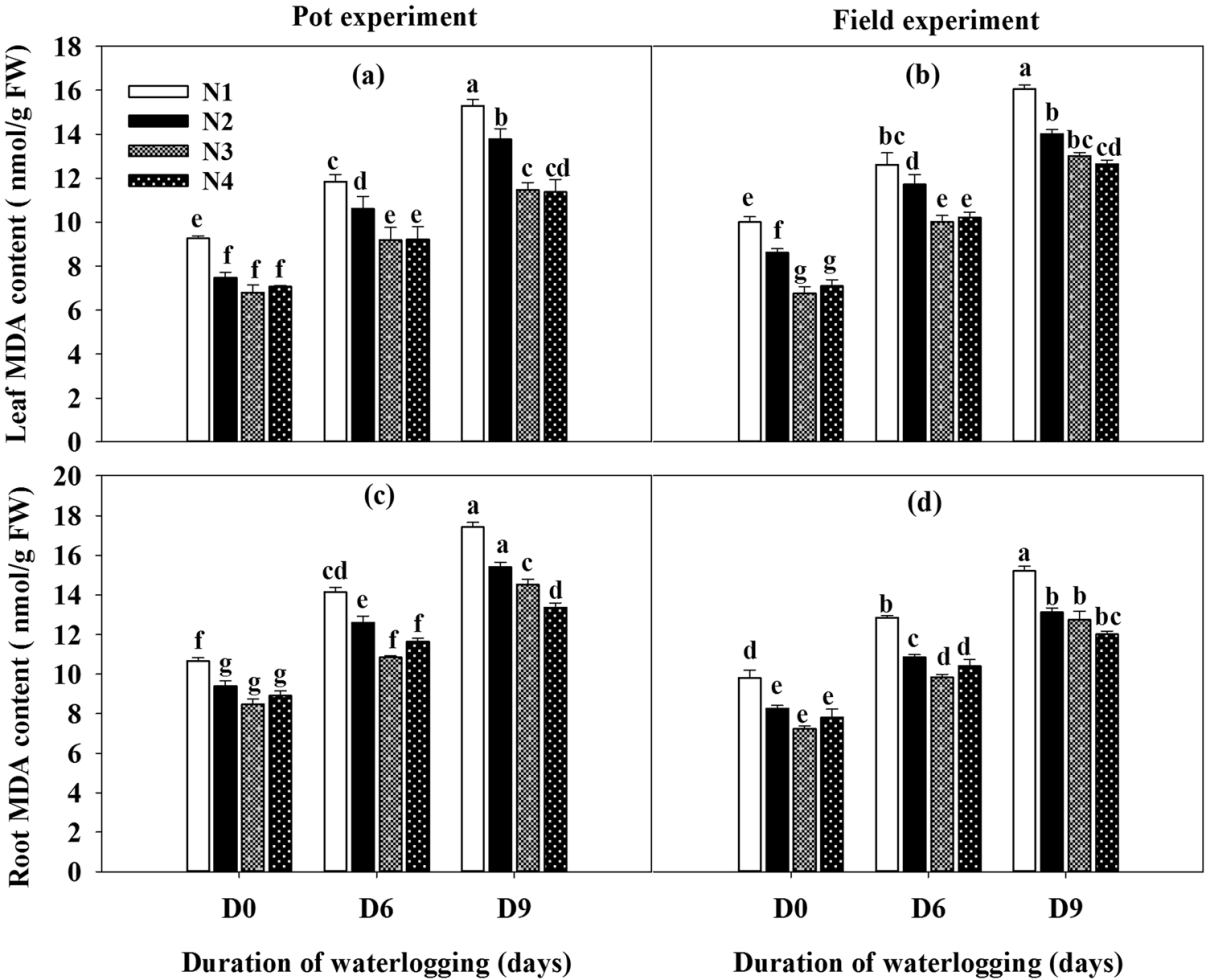
Influence of duration of waterlogging and nitrogen application on the production of malonaldehyde (MDA) of rape leaves and roots under pot and field experiments. Capped bars above means represent ±SE of three replicates. Small alphabetical letters above mean denote the significant differences among treatment at P ≤ 0.05. The different application levels of nitrogen were N1 (0 kg ha^−1^), N2 (30 kg ha^−1^), N3 (60 kg ha^−1^) and N4 (90 kg ha^−1^). D0, refers to without waterlogging; D6, refers to waterlogging for 6 days and D9, refers to waterlogging for 9 days.

Malonaldehyde (MDA) is the production of lipid peroxidation that damages enzymes and plant membranes. The waterlogged plants had higher MDA contents that varied with different duration of waterlogging in leaves and roots under the pot and field experiments (Fig. 2). Moreover, the levels of membrane damage in leaves and roots were decreased with the supplemental application of nitrogen fertilizer. More MDA was accumulated in the treatment of no nitrogen application at the same duration of waterlogging. No significant difference in MDA content of leaves and roots was observed between D6N3 and D0N1 treatment under the pot and field experiments. Compared with no application of nitrogen (N1), the MDA contents were decreased in the range of 20.24–23.67% and 20.92–25.69% for D6N3 and D9N4 treatments, respectively. The concentration of MDA in leaves and roots in both experiments was decreased with the lower duration of waterlogging and the higher level of nitrogen application.

### Activities of enzymatic antioxidants

**Figure 3:**
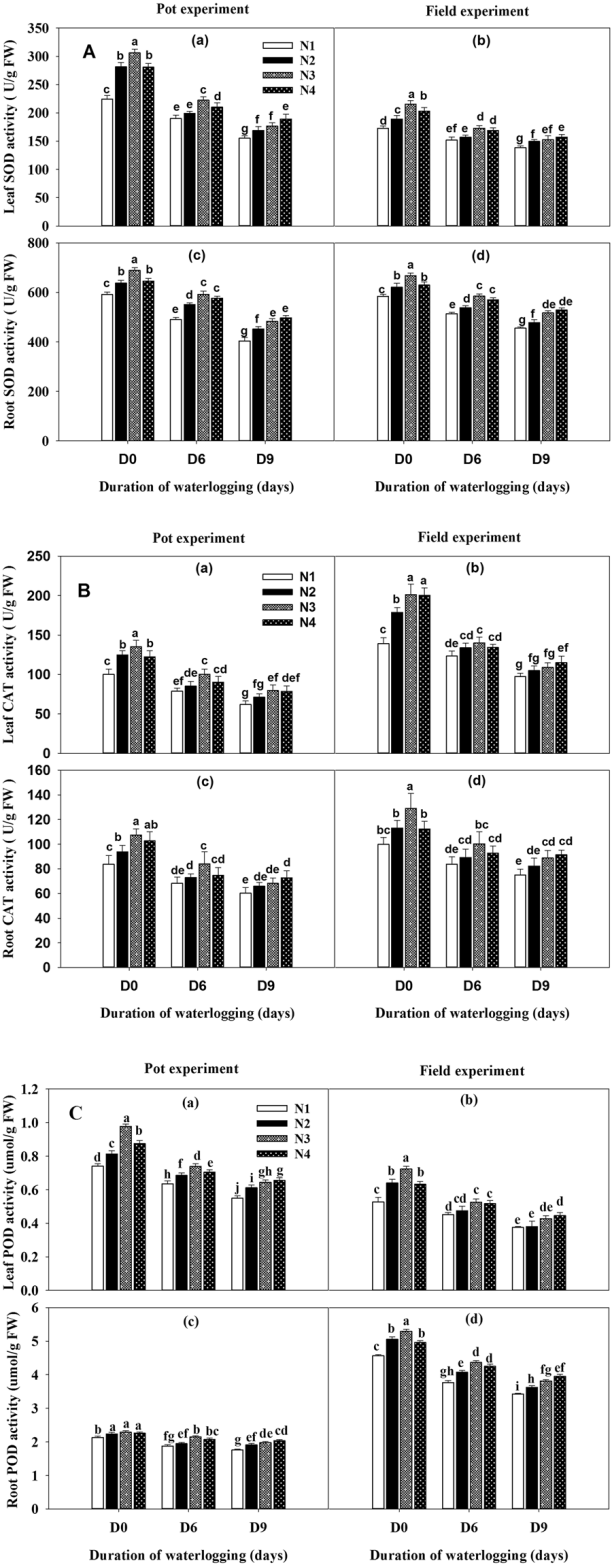
Influence of duration of waterlogging and nitrogen application on the activities of (**A**) superoxide dismutase (SOD), (**B**) catalase (CAT) and (**C**) peroxidase (POD) of rape leaves and roots under pot and field experiments. Capped bars above means represent ±SE of three replicates. Small alphabetical letters above mean denote the significant differences among treatment at P ≤ 0.05. The different application levels of nitrogen were N1 (0 kg ha^−1^), N2 (30 kg ha^−1^), N3 (60 kg ha^−1^) and N4 (90 kg ha^−1^). D0, refers to without waterlogging; D6, refers to waterlogging for 6 days and D9, refers to waterlogging for 9 days.

The activities of antioxidants enzymes (SOD, CAT, and POD) in the leaves and roots were considerably reduced after plants were subjected to waterlogging at the five-leaf stage in the both (pot and field) experiments, but the supplemental nitrogen application enhanced the activities of SOD, CAT, and POD (Fig. 3). The nitrogen fertilized plants had higher activities of enzymatic antioxidants under normal as well as waterlogged conditions. Under the D6N4 conditions, the SOD, CAT, and POD activities were increased significantly, in the range of 13.28–26.98% in leaves and roots, relative to the D6N1 treatment. No significant difference in SOD, CAT, and POD content of leaves and roots was observed between D6N3 and D0N1 treatment under the pot and field experiment. However, activities of SOD, CAT, and POD were significantly increased with the increase of nitrogen fertilizer application under waterlogged for nine days (D9), the maximum values were recorded at D9N4, although the values were still lower than the no waterlogged plants. It indicated that the application of nitrogen could improve the growth of waterlogged plants, and it was possible for plants to recover completely from waterlogging damage.

### Dry matter accumulation

The dry matter accumulation of rapeseed shoots (Table 1) and roots (Table 2) was considerably reduced after plants were subjected to waterlogging for 6 days and 9 days at the five-leaf stage under the pot and field experiments. Under the same duration of waterlogging, the application of N significantly increased the dry matter accumulation of root and shoot in rapeseed at different growth stages (except waterlogging 0 day), in the range of 4.21–63.17% at budding stage, 11.18–92.39% at flowering stage, and 16.70–70.73% at maturity stage, respectively. Under the 6-day waterlogging stress (D6), the accumulation of dry matter in rapeseed increased significantly with the increase of nitrogen application when the amount of nitrogen fertilizer was less than N3 (60 kg ha^−1^), while no significant difference of dry matter accumulation was observed between N3 and N4. However, the accumulation of dry matter was significantly increased with the increase of nitrogen fertilizer application under waterlogged conditions for nine days (D9), the maximum values were recorded at D9N4, although the values were still lower than the normal (no waterlogging) plants.

**Table 1:** Effects of waterlogging at the seedling stage and nitrogen application on the accumulation of dry matter in the shoot of winter rapeseed.

Water logging	N level	Pot experiment (g/plant)				Field experiment (kg/hm^2^)			
		0day	Budding Stage	Flowering Stage	Maturity Stage	0day	Budding Stage	Flowering Stage	Maturity Stage
D0	N1	3.41 ± 0.10^a^	12.90 ± 0.53^c^	40.89 ± 2.67 ^cd^	41.34 ± 1.32^e^	310.33 ± 12.14^ab^	1591.67 ± 37.31^c^	3977.00 ± 268.97 ^cd^	5201.00 ± 309.31^de^
	N2	3.53 ± 0.02^a^	14.73 ± 0.24^a^	45.47 ± 2.66^bc^	52.71 ± 3.92^c^	313.83 ± 5.78^a^	1775.00 ± 39.09^a^	4543.38 ± 302.14^abc^	7907.00 ± 380.72^b^
	N3	3.46 ± 0.10^a^	13.86 ± 0.24^b^	52.60 ± 2.82^a^	70.58 ± 1.25^a^	291.67 ± 6.92^abc^	1836.50 ± 29.77^a^	4849.00 ± 153.95^a^	8737.00 ± 349.93^a^
	N4	3.43 ± 0.22^a^	13.44 ± 0.42^bc^	50.62 ± 2.54^ab^	61.78 ± 0.74^b^	315.83 ± 12.68^a^	1681.50 ± 35.06^b^	4623.67 ± 258.83^ab^	6267.00 ± 110.78^c^
D6	N1	2.64 ± 0.04^b^	9.41 ± 0.09 ^f^	32.08 ± 0.10^ef^	30.24 ± 1.47^fg^	280.67 ± 12.23^bcd^	926.50 ± 18.50 ^h^	2328.50 ± 39.91 ^g^	3036.00 ± 220.46^fg^
	N2	2.62 ± 0.13^b^	11.33 ± 0.11^de^	36.98 ± 0.50^de^	35.29 ± 1.48 ^f^	257.33 ± 8.59^d^	1154.83 ± 30.08 ^f^	3396.83 ± 198.82^ef^	4543.50 ± 210.91^e^
	N3	2.51 ± 0.16^b^	14.00 ± 0.17^ab^	45.05 ± 3.12^bc^	46.64 ± 0.86^d^	272.33 ± 8.67 ^cd^	1392.33 ± 23.64^d^	4116.83 ± 127.68^bcd^	5495.50 ± 129.01^d^
	N4	2.44 ± 0.09^b^	11.78 ± 0.20^d^	40.95 ± 2.12 ^cd^	40.96 ± 0.63^e^	269.33 ± 11.57 ^cd^	1283.00 ± 34.88^e^	3738.67 ± 189.05^de^	5243.50 ± 94.91^de^
D9	N1	2.00 ± 0.15^c^	6.55 ± 0.06 ^h^	18.39 ± 1.19 ^g^	23.75 ± 1.20 ^h^	172.67 ± 13.72^e^	577.00 ± 15.60^j^	1718.00 ± 20.23 ^h^	2062.50 ± 180.60 ^h^
	N2	2.09 ± 0.04^c^	8.07 ± 0.07 ^g^	29.61 ± 1.49 ^f^	28.27 ± 1.34^gh^	155.17 ± 5.78^e^	757.00 ± 23.88^i^	2217.33 ± 137.51^gh^	2740.00 ± 201.73 ^g^
	N3	2.05 ± 0.03^c^	9.34 ± 0.27 ^f^	34.28 ± 1.99^def^	31.14 ± 0.21^fg^	148.50 ± 10.75^e^	1024.17 ± 34.61 ^g^	2963.17 ± 143.89 ^f^	3171.50 ± 31.28^fg^
	N4	2.01 ± 0.05^c^	10.69 ± 0.15^e^	35.39 ± 2.71^def^	34.71 ± 1.76 ^f^	163.00 ± 6.66^e^	1104.67 ± 15.61^fg^	3108.17 ± 175.51 ^f^	3707.00 ± 263.51 ^f^

Values are means of three replicates ±SE. Values followed by the similar lower case letters within columns for waterlogging treatments don’t differ significantly according to Duncan’s test (P ≤ 0.05).The different application levels of nitrogen were N1 (0 kg ha^−1^), N2 (30 kg ha^−1^), N3 (60 kg ha^−1^) and N4 (90 kg ha^−1^). D0, refers to without waterlogging; D6, refers to waterlogging for 6 days and D9, refers to waterlogging for 9 days. 0 day refers to the days after nitrogen application.

**Table 2:** Effects of waterlogging at the seedling stage and nitrogen application on the accumulation of dry matter in the root of winter rapeseed.

Water logging	N level	Pot experiment (g/plant)				Field experiment (kg/hm^2^)			
		0day	Budding Stage	Flowering Stage	Maturity Stage	0day	Budding Stage	Flowering Stage	Maturity Stage
D0	N1	0.44 ± 0.03^a^	3.63 ± 0.05^b^	6.58 ± 0.11^c^	7.47 ± 0.11^e^	41.42 ± 3.96^ab^	371.67 ± 11.18^b^	626.33 ± 13.72^b^	780.50 ± 9.85^c^
	N2	0.45 ± 0.02^a^	4.63 ± 0.13^a^	7.61 ± 0.09^b^	10.66 ± 0.31^c^	46.00 ± 3.88^a^	475.00 ± 78.49^a^	790.00 ± 24.27^a^	1180.00 ± 31.82^b^
	N3	0.43 ± 0.01^a^	4.50 ± 0.08^a^	8.02 ± 0.25^a^	12.71 ± 0.17^a^	40.00 ± 2.52^ab^	489.17 ± 17.73^a^	766.17 ± 8.17^a^	1277.00 ± 37.97^a^
	N4	0.42 ± 0.01^a^	4.45 ± 0.03^a^	7.94 ± 0.05^ab^	11.11 ± 0.09^b^	45.17 ± 9.68^a^	465.50 ± 21.30^a^	753.33 ± 25.13^a^	1158.50 ± 35.38^b^
D6	N1	0.34 ± 0.01^b^	2.41 ± 0.07^e^	5.08 ± 0.07^e^	5.43 ± 0.05 ^h^	32.33 ± 1.30^bc^	256.00 ± 26.10^def^	418.33 ± 11.09^d^	476.50 ± 17.52^fg^
	N2	0.33 ± 0.01^b^	3.20 ± 0.05^c^	5.92 ± 0.13^d^	6.47 ± 0.16 ^f^	27.17 ± 0.88^cde^	300.33 ± 16.10^bcd^	481.17 ± 10.17^c^	658.00 ± 34.97^d^
	N3	0.32 ± 0.02^b^	3.73 ± 0.04^b^	6.69 ± 0.06^c^	7.97 ± 0.08^d^	32.67 ± 1.36^bc^	379.17 ± 8.08^b^	638.67 ± 15.28^b^	822.00 ± 21.70^c^
	N4	0.30 ± 0.01^bc^	3.71 ± 0.13^b^	6.11 ± 0.03^d^	6.56 ± 0.04 ^f^	28.17 ± 1.30 ^cd^	356.50 ± 12.77^bc^	622.67 ± 8.80^b^	762.50 ± 11.95^c^
D9	N1	0.23 ± 0.02^d^	1.73 ± 0.08 ^g^	3.12 ± 0.10 ^g^	3.84 ± 0.11^j^	23.17 ± 2.20^cde^	183.50 ± 9.73 ^f^	257.17 ± 10.65 ^f^	302.00 ± 20.90 ^h^
	N2	0.26 ± 0.02 ^cd^	2.13 ± 0.05 ^f^	4.55 ± 0.07 ^f^	4.76 ± 0.08^i^	18.17 ± 1.20^d^	207.33 ± 21.18^ef^	340.33 ± 3.56^e^	423.00 ± 17.90 ^g^
	N3	0.25 ± 0.01 ^cd^	2.57 ± 0.07^de^	5.14 ± 0.12^e^	5.58 ± 0.10^gh^	16.50 ± 1.04^e^	270.17 ± 14.42^cdef^	421.67 ± 11.92^d^	542.50 ± 16.26^ef^
	N4	0.23 ± 0.01^d^	2.71 ± 0.03^d^	5.21 ± 0.15^e^	5.91 ± 0.08 ^g^	18.50 ± 2.36^de^	290.83 ± 9.28^bcde^	455.83 ± 6.77 ^cd^	585.50 ± 15.88^e^

Values are means of three replicates ±SE. Values followed by the similar lower case letters within columns for waterlogging treatments don’t differ significantly according to Duncan’s test (P ≤ 0.05).The different application levels of nitrogen were N1 (0 kg ha^−1^), N2 (30 kg ha^−1^), N3 (60 kg ha^−1^) and N4 (90 kg ha^−1^). D0, refers to without waterlogging; D6, refers to waterlogging for 6 days and D9, refers to waterlogging for 9 days. 0 day refers to the days after nitrogen application.

### Nitrogen accumulation

**Figure 4:**
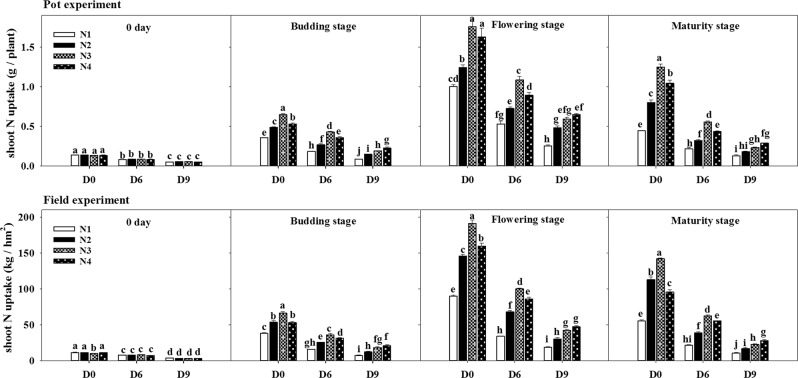
Influence of duration of waterlogging and nitrogen application on the accumulation of nitrogen in rape shoots in pot and field experiments. Capped bars above means represent ±SE of three replicates. Small alphabetical letters above mean denote the significant differences among treatment at P ≤ 0.05. The different application levels of nitrogen were N1 (0 kg ha^−1^), N2 (30 kg ha^−1^), N3 (60 kg ha^−1^) and N4 (90 kg ha^−1^). D0, refers to without waterlogging; D6, refers to waterlogging for 6 days and D9, refers to waterlogging for 9 days.0 day refers to the days after nitrogen application.

The accumulation of nitrogen in the rapeseed shoots and roots was reduced after plants were subjected to waterlogging at the five-leaf stage under the pot and field experiments (Fig. 4). The negative effects were varied with the plant’s growth stages and the duration of waterlogging. The application of nitrogen fertilizer increased the accumulation of nitrogen of rapeseed in shoots at various growth stages. With the growth process of plants, the nitrogen content of rapeseed shows a trend of increasing and then decreasing. When the amount of nitrogen fertilizer was less than N3 (60 kg ha^−1^), the accumulation of nitrogen in rapeseed was significantly increased with increasing the nitrogen application but no significant difference was observed between D6N3 and D6N4 under waterlogged for 6 days. However, the nitrogen concentration was significantly increased with the increase of nitrogen fertilizer application under waterlogged for nine days (D9), although the values were still lower than plants without waterlogging stress, and the maximum values were recorded at D9N4. The most significant effect of nitrogen fertilizer application was observed on the D0N3, D6N3 and D9N4 treatments at different stages of growth. Additionally, the higher accumulation of nitrogen in shoots was recorded in pot experiment.

## Discussion

Waterlogging treatments significantly affects the photosynthesis, antioxidant enzyme activity, inhibited the growth of the above-ground parts of the crops, nitrogen uptake, and longer the duration of waterlogging^26,27^. Waterlogging was highly detrimental to the plants because of severe damage and the inability to initiate new tillers under waterlogging and adequately resume root growth during the recovery^28^. The activities of enzymes, membrane lipid peroxidation and leaf photosynthesis of rapeseed changed under the stress of waterlogging, which affected the growth and development of rapeseed^29^. However, waterlogging stress significantly inhibited the growth and development of rapeseed but the application of nitrogen fertilizer effectively reduced the damage of waterlogging stress. Chlorophyll is a main pigment for photosynthesis of plants, which is most important for plant physiological processes^19^. Nitrogen is the main component of chloroplasts, and the effect of nitrogen on chloroplasts restricts photosynthesis^30^. In this study, waterlogging at the seedling stage of rapeseed plants significant decreases the leaf chlorophyll content. Moreover, chlorophyll concentration was increased with the lower duration of waterlogging and the higher level of supplemental nitrogen application in both experiments (Fig. 1). After waterlogging at the seedling stage, soil nitrogen was leached to the depth of the soil in the form of NO_3_-N, and the available mineral nitrogen concentration in the soil was reduced. So that, the insufficient nitrogen fertilizer leads to poor chloroplast structure in the mesophyll cells^20^. Some previous reports also confirmed that the relatively low photosynthetic capacity of waterlogged plants can be attributed to decreased leaf chlorophyll content^31–33^ and reduced the activity of photosynthesis-related proteins^1^, thereby reducing dry matter accumulation and nutrient uptake in crops. Furthermore, compared with no fertilizer-treated plants, the application of nitrogen fertilizer significantly increased the chlorophyll content. After 6 days of waterlogging stress, the application of nitrogen fertilizer at 60 kg ha^−1^ restored the total chlorophyll content of the rapeseed seedlings. Our results indicated that nitrogen application can alleviate the degradation of photosynthetic pigments caused by waterlogging stress to a certain extent, thereby improving the photosynthesis ability of plants and contribute to the accumulation of dry matter. However, long-term waterlogging stress can destroy the chloroplast structure of seedlings, thus the synthesis of photosynthetic pigments cannot be fully restored.

In this study, waterlogging at the seedling stage of rapeseed plants significantly increases the MDA content of leaves and roots (Fig. 2). The enhanced lipid peroxidation in plants as MDA accumulation leads to the oxidative damage caused by ROS^34^. However, the application of nitrogen fertilizer significantly decreased the content of MDA. The higher accumulation of MDA seems to be associated with an impaired ability for H_2_O_2_ scavenging^35^. In addition, the application of nitrogen fertilizer cannot restore the MDA content to natural level when the plant was exposed to water stress for 9 days. This might be due to the damage of plants after nine days of waterlogging stress which exceeded the metabolic balance system, and slowed down the degree of peroxidation of cell membranes, or it is related to insufficient nitrogen application after long-term waterlogging. Therefore, the optimal application level of nitrogen after long-term waterlogging stress should be further studied.

Plants hold the efficient defense system with enzymatic and non-enzymatic antioxidant to cope with oxidative stress induced by ROS^36,37^. In the present study, the application of nitrogen fertilizer increased the activities of antioxidant defense systems in rapeseed leaves and roots. Waterlogging stress limited the enzymatic antioxidative defense system; however, the activities of antioxidants in leaves and roots such as SOD, POD, and CAT were reduced with the longer duration of waterlogged (Fig. 3). Moreover, the results of both experiments showed similar response to waterlogging stress. A key role of antioxidants in waterlogging tolerance has also been reported in mungbean plants^38^. The experimental results indicated that the application of nitrogen after waterlogging stress may induce the expression of related antioxidant enzyme genes, improve the physiological indexes of plants and timely remove the active oxygen generated by waterlogging stress, which promotes the recovery of physiological metabolic activities of plants.

The accumulation of dry matter in shoots and roots of rapeseed were significantly reduced when plants faced the waterlogging stress conditions at the seedling stage, but the application of nitrogen fertilizer alleviated the plant damage caused by waterlogging (Tables 1 and 2). During the seedling stage, waterlogging is a serious physiological constraint and there is a significant relationship between yield and growth of plant during this stage^39^. Previous studies showed that waterlogging after flowering stage significantly reduced the 1000-grain weight and dry matter quality of rapeseed, among which the dry matter quality decreased by 25.6%, and the 1000-grain weight decreased by 12.3%^29^. The present experiments confirmed that the physiological function of rapeseed plants was retarded during the time of waterlogging, and its adverse effects remained afterward, resulting in a highly significant decrease of dry matter and nitrogen accumulation in the maturity stage^33^. Waterlogging significantly inhibited the accumulation of dry matter in rapeseed and affected the transfer of photosynthetic products to kernels and the formation of grain yield^36^. After waterlogging stress, the application of nitrogen fertilizer at the seedling stage increased the dry matter content of rapeseed shoots and roots and improved the growth condition of waterlogged rapeseed plants^40,41^. However, long-term waterlogging stress caused serious damage to plant seedlings, and the supplemental nitrogen application could not return to the natural level. This may be due to 9-day flooding stress that damages the plant beyond its own maintainable balance or is associated with insufficient nitrogen application after long-term waterlogging. Nutrients are the most important factors affecting the actual yield and maximum yield potential of crop, except for the limiting factor of moisture^42^. Generally, the plant’s growth and productivity affected by the assimilation, acquisition, and distribution of N in various organs of the plants^43^. After waterlogging in the seedling stage, soil nitrogen is leached to the depth of the soil in the form of NO_3_-N, and the available mineral nitrogen accumulation in the soil is reduced, so the nutrient absorbed and utilized are reduced by the root^44^. Studies have shown that, the oat species suffered from the severe damage during waterlogging, and the uptake of nitrogen and the N-concentration of shoots were reduced after 7 days, tiller initiation and root growth after 14 days, and shoot growth after 21 days^35^. In the present study, decreasing trend was observed regarding the nitrogen accumulation of rapeseed in shoots with the increase in waterlogging stress levels, nonetheless, such waterlogged-induced adversities were more prominent in D9 than D6 (Fig. 4). Furthermore, compared with no fertilizer-treated plants, the application of nitrogen fertilizer significantly increased the content of nitrogen in shoots of rapeseed.

## Materials and Methods

### Plant material and experimental site

The seeds of widely grown winter rapeseed cultivar (Chuanyou36) were obtained from the Crop Research Institute, Sichuan Academy of Agricultural Sciences, China. The experiment soil (field and pot experiments) contained 31.80 g kg^−1^ organic matter, 17.16 g kg^−1^ total nitrogen, 199.50 mg kg^−1^ available nitrogen, 22.50 mg kg^−1^ available phosphorous, 66.50 mg kg^−1^ available potassium and 6.50 pH.

### Pot experiment

The pot experiment was carried out in the greenhouse at the Soil and Fertilizer Research Institute, Sichuan Academy of Agricultural Sciences, China (longitude 104°11′E, latitude 30°62′N) during 2015 and 2016. The soil was taken from the experimental field soil (0–20 cm). The total weight of each pot was 7 kg after filling with air-dried soil. Fertilizers were applied at the equivalent of 0.08 g kg^−1^ N, 0.04 g kg^−1^ P_2_O_5_, 0.05 g kg^−1^ K_2_O and 1.43 mg kg^−1^ B, and thoroughly mixed in the soil of each pot. Punch 3 circles at 5, 15, and 25 cm away from the bottom of the plastic basin, with the pore size of about 1 cm, and affix the filter screen. The plastic basins with different treatments were placed in different water tanks, and the water surface was 1 cm higher than the soil surface during the waterlogging treatment. Two plants were maintained in each pot. At the five-leaf stage, waterlogging treatments were imposed on the plants. First, the waterlogging treatment was carried out for nine days (D9), and at the same time after three days, the waterlogging treatment was started for six days (D6), and with no waterlogging treatment (D0) was as the control treatment. At the end of waterlogging treatment, the water in the water tank was released, so that the soil water content in the plastic basin was 70–80% of the field water capacity, return to normal water management. Three days after normal water management was resumed, four different application levels of nitrogen were applied to three different waterlogging stress treatments, i.e., N1 (0 kg ha^−1^), N2 (30 kg ha^−1^), N3 (60 kg ha^−1^) and N4 (90 kg ha^−1^).The treatments were arranged in a completely randomized design (CRD). Each treatment was replicated thrice, and there were six pots per replicate.

### Field experiment

The field experiment was conducted during 2015 and 2016 at the Liyan, Guanghan, Sichuan, China (longitude 104°17′E, latitude 31°04′N). The area of each plot was 4 m×5 m and separated by the plastic film as water barriers. For avoiding water side leakage, every plastic film was buried 1.0 m below the surface and the distance between each plot was 1.5 meters. Fertilizer was applied before sowing, containing N 180 kg ha^−1^, P_2_O_5_ 90 kg ha^−1^, K_2_O 117 kg ha^−1^, and B 3 kg ha^−1^, respectively. The rapeseed was sown on 20 September with the plant density of 15.0 × 10^4^ plant ha^−1^ and allowed to grow under normal conditions up to the five-leaf stage. After that, waterlogging treatments were imposed on the plants for 6 days (D6), 9 days (D9), and no waterlogging (D0) was imposed as control treatment. During the waterlogging treatments, the water was maintained at 1 cm above the soil surface. In the control (D0) treatment, soil moisture was kept at 70–80% of field capacity during the whole growth period. At the end of waterlogging treatment, ditches were opened for drainage in each plot, so that the soil moisture content maintained 70–80% of the field water capacity and normal water management could be restored. Three days after normal water management was resumed, four different application levels of nitrogen were applied to three different waterlogging stress treatments, i.e., N1 (0 kg ha^−1^), N2 (30 kg ha^−1^), N3 (60 kg ha^−1^) and N4 (90 kg ha^−1^). Each treatment was replicated three times in a randomized complete block design (RCBD).

### Photosynthetic pigments and antioxidant activity

In pot and field experiments, fully expanded, healthy and undamaged plant leaves (third from the top of new leaf) and the roots (fibrous roots near apex) from each replication were sampled at 20 days after the application of different nitrogen fertilizer to measure the chlorophyll concentrations and other biochemical analyses. After washing with double distilled water, the total Chlorophyll content was determined according to Peng and Liu^45^. The extraction of 250 mg leaf without vein (leaf blade) was done with 10 ml ethanol-acetone (vol. 1:2), and the extract was transferred to a 15 ml tube. The tubes were placed in the dark to avoid light for 24 hours. The absorbance was measured at 645 nm, 663 nm, and 652 nm. The chlorophyll content was computed by the following formulae:

$${\rm{Chlorophyll}}\,{\rm{a}}\,{\rm{content}}\,({\rm{mg}}\,{{\rm{g}}}^{-1}{\rm{tissue}})=(12.7{\rm{D}}663-2.69{\rm{D}}645)\times {\rm{V}}/(1000\times {\rm{W}})$$

$${\rm{Chlorophyll}}\,{\rm{b}}\,{\rm{content}}\,({\rm{mg}}\,{{\rm{g}}}^{-1}{\rm{tissue}})=(22.7\,{\rm{D}}645-4.68{\rm{D}}663)\times {\rm{V}}/(1000\times {\rm{W}})$$

$${\rm{Total}}\,{\rm{chlorophyll}}\,({\rm{mg}}\,{{\rm{g}}}^{-1}{\rm{tissue}})={\rm{D}}652\times {\rm{V}}/(34.5\times {\rm{W}})/{\rm{Chl}}\,{\rm{a}}+{\rm{Chl}}\,{\rm{b}}$$

where, D663, D645, and D652 respectively are the corresponding wavelengths of the light density value, V is extracting liquid volume, and W is leaf fresh weight.

Lipid peroxidation of rapeseed leaves and roots were determined as MDA content, measured by thiobarbituric (TBA) method^46^. Lipid hydroperoxide decomposition products can condense with thiobarbituric acid (TBA) to produce red compounds. The absorbance for MDA was measured at 532 nm and expressed as nmol/g fresh weight.

The crude extract was obtained by homogenization of the plant material in the extraction buffer at 4 °C (weighing 0.5 g of plant material and phosphate buffer 6 ml, pH 7.8). The extract was stored in an ultra-low temperature freezer for the determination of SOD, POD, CAT activity. Superoxide dismutase (SOD) activity and peroxidase (POD) were analyzed by the Wang method^46^. SOD activity was measured through the inhibition of NBT light reduction (3000 lux for 10 min). POD activity was measured through the absorbance of the reaction mixture containing 100 ml enzyme extract, 50 mM phosphate buffer (pH 7.0), 28 ml guaiacol and 19 ml H_2_O_2_ was read at 420 with a 30 s interval up to 2 min and used the absorbance change 0.01 as a POD activity. Catalase (CAT) activity was analyzed by the hydrogen peroxide reduction method^46^. Leaf extracts were added to 5 ml 0.1 N H_2_O_2_ and kept at 20 °C for 5 min, then 1 ml 20% KI solution, 3 drops of 10% (NH4)_6_Mo_7_O_24_ solution and 5 drops of 1% starch solution were added. The reaction solution was titrated with 0.02 N Na_2_S_2_O_3_ until the disappearance of the blue color. CAT activity was determined through the reduction of H_2_O_2_ during the given period.

### Dry matter and nutrient accumulation

Rapeseed plants were sampled at 0 day after waterlogging, budding stage, flowering stage, and maturity stage after the supplemental nitrogen application imposition. To examine the accumulation of dry matter and nutrients, six plants per treatment were randomly selected for each sampling and harvested at different growth stages. The plants were excavated with roots and the roots were cut from the cotyledonary nodes at sampling. Shoots and roots were thoroughly washed with distilled water and were oven-dried at 105 °C for 30 min and later at 80 °C until constant weight. Dry samples of 300 mg were digested with 8 ml sulfuric acid (H_2_SO_4_) in a sealed chamber. N concentration in shoots and roots was determined using the Kjeldahl method^47^. The Nitrogen accumulation was computed by the following formulae:

$${\rm{N}}\,{\rm{accumulation}}\,({\rm{kg}}\,{{\rm{ha}}}^{-1})={\rm{dry}}\,{\rm{matter}}({\rm{kg}}\,{{\rm{ha}}}^{-1})\times {\rm{N}}\,{\rm{concentration}}\,( \% )/100$$

### Statistical analysis

Data were subjected to the analysis of variance (ANOVA) technique for statistical analysis using SPSS 17.0 software (SPSS, Chicago, IL, USA) and differences among treatments were tested according to Duncan’s multiple range test at 5% level of significance and the SigmaPlot 13.0 software (Systat Software Inc., San Jose, CA, USA) was used for the presentation of data in figures.

## Conclusions

In summary, waterlogging stress significantly inhibited the growth and development of rapeseed, nonetheless, the application of nitrogen fertilizer could effectively alleviate the damage of waterlogging. The supplemental nitrogen application increased the chlorophyll content in leaves, enhanced the activities of SOD, CAT and POD, and decreased the MDA content in leaves and roots of the rapeseed. And the application of nitrogen fertilizer after waterlogging increased the accumulation of dry matter and nitrogen contents in rapeseed at different growth stages. Under D6N3 and D9N4 conditions, the chlorophyll contents, antioxidant enzyme activity of leaves and roots increased significantly, and MDA content decreased significantly, compared with waterlogging without nitrogen application. However, the application of nitrogen fertilizer cannot restore the plant to a natural level when the plant was exposed to waterlogging stress for 9 days. Consequently, in the main planting region of rapeseed, the application of 60 kg ha^−1^ nitrogen fertilizer after 6 days waterlogging stress or 90 kg ha^−1^ nitrogen fertilizer after 9 days waterlogging stress had significantly enhanced the physiological and metabolic activities of rapeseed and contribute to the better recovery of rapeseed growth.
